# The Kynurenine Pathway in Obese Middle-Aged Women with Normoglycemia and Type 2 Diabetes

**DOI:** 10.3390/metabo12060492

**Published:** 2022-05-29

**Authors:** Justyna Kubacka, Magdalena Staniszewska, Ilona Sadok, Grazyna Sypniewska, Anna Stefanska

**Affiliations:** 1 Department of Laboratory Medicine, Collegium Medicum, Bydgoszcz, Nicolaus Copernicus University, 87-110 Torun, Poland; justynkakubacka@wp.pl (J.K.); odes@cm.umk.pl (G.S.); 2 Laboratory of Separation and Spectroscopic Method Applications, Centre for Interdisciplinary Research, Faculty of Science and Health, The John Paul II Catholic University of Lublin, Konstantynów 1J, 20-708 Lublin, Poland; magdalena.staniszewska@kul.pl (M.S.); ilona.sadok@kul.pl (I.S.)

**Keywords:** tryptophan, kynurenine pathway, type 2 diabetes, obese women, kynurenic acid, 3-hydroxykynurenine

## Abstract

We examined the relationships of tryptophan (Trp) and the metabolites of the kynurenine pathway (KP) to the occurrence of type 2 diabetes (T2D) and metabolic risk factors in obese middle-aged women. The study included 128 obese women divided into two subgroups: a normoglycemic group (NG, *n* = 65) and a T2D group (*n* = 63). The concentrations of serum tryptophan (Trp), kynurenine (Kyn), 3-hydroxykynurenine (3HKyn), quinolinic acid (QA), and kynurenic acid (Kyna) were analyzed using ultra-high-performance liquid chromatography coupled with electrospray ionization/triple quadrupole mass spectrometry. Blood biochemical parameters and anthropometric parameters were measured. The women with T2D had significantly higher Trp, Kyna, Kyna/QA ratio, and Kyna/3HKyn ratio values than the NG women. Logistic regression analysis showed that the concentrations of Trp and Kyna and the values of the Kyna/3HKyn ratio were most strongly associated with T2D occurrence, even after controlling for confounding factors. The model with Trp level and Kyna/3HKyn ratio accounted for 20% of the variation in the presence of T2D. We also showed a different pattern of correlations between kynurenines and metabolic factors in the NG and T2D women, which was mostly reflected in the stronger relationship between BMI and KP metabolites in the NG obese women. An increase in Trp and Kyna levels with an accompanying increase in Kyna/3HKyn ratio value is associated with the occurrence of T2D in obese middle-aged women.

## 1. Introduction

**Figure 1:**
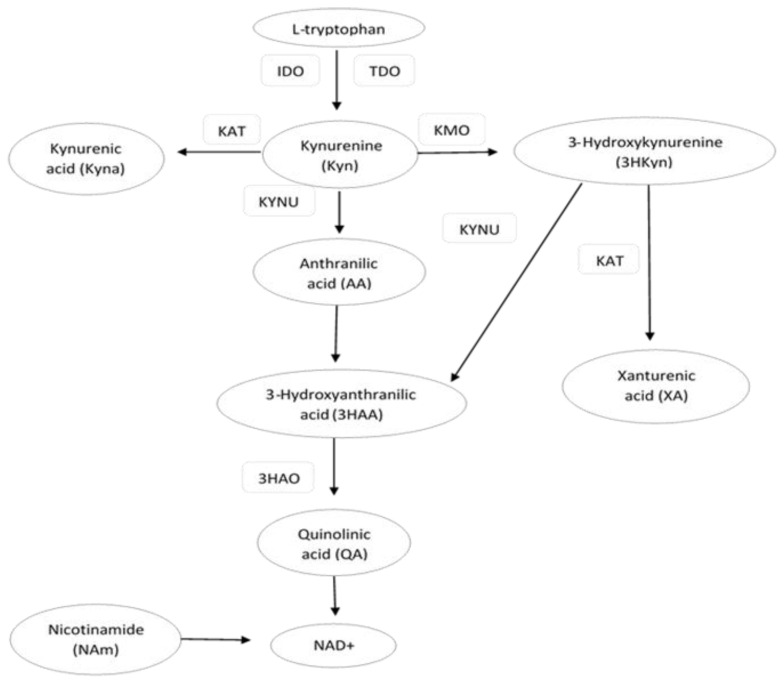
Schematic illustration of some major steps, metabolites, and substrates involved in the kynurenine pathway (KP). IDO—indoleamine 2,3-dioxygenase; TDO—tryptophan 2,3-dioxygenase; KAT—kynurenine aminotransferase; KMO—kynurenine-3-monooxygenase; 3HAO—3-hydroxyanthranilic acid dioxygenase; KYNU—kynureninase.

Tryptophan (Trp) is an aromatic essential amino acid that plays an important role in the metabolic processes of the human body. The main food sources with the highest Trp contents are meat, fish, chocolate, eggs, and legumes [1]. The kynurenine pathway (KP) is the major route of Trp catabolism. This pathway is initiated by indoleamine 2,3-dioxygenase (IDO) and tryptophan 2,3-dioxygenase (TDO), the enzymes that catalyze the conversion of Trp to kynurenine (Kyn). Kyn is further converted into the following KP metabolites (kynurenines): kynurenic acid (Kyna), 3-hydroxykynurenine (3HKyn), anthranilic acid (AA), and quinolinic acid (QA). 3Hkyn can be enzymatically converted to xanthurenic acid (XA) [2,3] (Figure 1). A dysregulated KP has been linked to various diseases, including neurodegenerative disorders, multiple sclerosis, depression, schizophrenia, cancer, and renal or hepatic disorders. For this reason, KP metabolites and enzymes are potential pharmacological and diagnostic targets for disease control and homeostasis [2,4,5,6,7,8,9]. An imbalance in the levels of circulating KP metabolites has also been reported in obesity and obesity-related metabolic disorders, such as cardiovascular disease (CVD) and type 2 diabetes (T2D) [10,11]. It is well known that obesity and inflammatory states are associated with a redirection of the KP in favor of the breakdown of Trp into downstream products reflected by increased serum Kyn levels [12,13,14,15]. The concentration of serum Trp and its metabolites, such as Kyn, Kyna, XA, 3HKyn, QA, and KP enzymes, were found to be associated with T2D [16,17,18]. However, the data on these metabolites in relation to T2D are often contradictory in human studies [19,20]. It is also known that the regulation of KP is affected by many factors, e.g., body mass, age, gender, and inflammatory state [12,21]. These different covariates may influence the results of human studies, especially in those with small sample sizes. For this reason, we decided to compare two groups of middle-aged obese women with T2D and normoglycemia (NG), which were matched for body mass and other possible confounding factors. We evaluated obese middle-aged women because they are more likely to have metabolic disturbances that increase the risk of T2D, which is partially associated with aging and the menopause [22]. Hormonal changes during the menopause are associated with the redistribution of adipose tissue accompanied with inflammatory changes that may affect the KP. The loss of estrogens may also affect the concentrations of Trp and KP metabolites [6]. Moreover, some earlier studies suggest that the serum concentrations of KP metabolites are lower in women and differ across different age categories, especially those below 40 and above 60 [23,24]. To the best of our knowledge, only one study has been published that refers to obese middle-aged European women from the ABOS cohort (Biological Atlas of Severe Obesity) [13]. In this study, however, the women presented with more severe obesity than in our group. We hypothesized that the concentrations of Trp and its KP metabolites are associated with the occurrence of T2D and metabolic risk factors in middle-aged obese women. A better understanding of the kynurenine pathway may be helpful in the assessment of diagnostic and therapeutic usefulness of the components of this pathway for cardiometabolic disturbances in middle-aged obese women.

## 2. Results

The characteristics of the study participants are shown in Table 1. The groups of women with and without T2D were matched for BMI, WC obesity categories, physical activity, menopausal status, smoking habits, and alcohol consumption but differed significantly in terms of age. Neither group differed significantly in terms of TSH or concentrations of proinflammatory markers (CRP, neopterin). Statistically significant differences in the values of glucose, HbA1c, HOMA-IR, HDL-C, TG, ALT, AST, GGT, and eGFR were observed between the groups.

The concentrations of Trp and KP metabolites are shown in Table 2. The levels of Trp and Kyna and the values of Kyna/QA and Kyna/3HKyn ratios were significantly higher in the women with T2D. All statistically significant differences in the values of KP metabolites remained significant after controlling for age.

Table 3 shows Spearman correlation coefficients between Trp and its KP metabolites (kynurenines), and the metabolic factors. We observed only weak or very weak correlations between the analyzed parameters. The Trp concentration correlated significantly with TG. The kynurenines and their ratios most strongly correlated with BMI, TG, ALT, GGT, creatinine, and eGFR. We also analyzed the correlation between Trp and KP metabolites in the overall study group. We found statistically significant (all *p* < 0.05) correlations between Trp and 3HKyn (r = 0.30), Kyn and QA (r = 0.34), 3HKyn and QA (r = −0.41), and Kyna and 3HKyn (r = −0.25).

The association between Trp and its metabolites with T2D was also examined in the logistic regression models (Table 4). The KP metabolites and their ratios that were significantly different between the NG and T2D women were included separately in logistic regression models as independent variables. We found that all unadjusted models were statistically significant. The values of Trp, Kyna, Kyna/3HKyn ratio, and Kyna/QA ratios were positively associated with T2D occurrence.

The models for Trp, Kyna, and Kyna/3HKyn were statistically significant after adjusting for age, BMI, eGFR values, GGT concentration, and hypertriglyceridemia. According to the Negelkerke R-squared, Trp accounted for 7.4% of the variation in the presence of T2D in the unadjusted model. Kyna, Kyna/3HKyn ratio, and Kyna/QA ratio had values of the Negelkerke R-squared in the unadjusted models of 7.5%, 8.3%, and 6.1%, respectively. The models with Trp (*p* = 0.006) and Kyna (*p* = 0.01) as independent variables accounted for 15% of the variation in the presence of T2D according to the Negelkerke R-squared, while the model with Trp (*p* = 0.001) and Kyna/3HKyn ratio (*p* = 0.001) accounted for 20% of the variation in the presence of T2D. 

Finally, we analyzed the Spearman correlation coefficients separately in the NG and T2D obese women to evaluate if the relationship between KP metabolites and metabolic factors depends on glycemic status (Table 5). We observed a different pattern of correlations in the NG and T2D women.

The values of BMI and WC were only significantly correlated with individual KP metabolites in the NG women. The CRP concentration correlated with Kyna and 3HKyn in the NG women, while the neopterin concentration had a tendency to correlate with 3HKyn in the T2D women. We also analyzed correlations between Trp and KP metabolites separately in the NG and T2D women. We found statistically significant correlations (all *p* < 0.05) between Trp and 3HKyn (r = 0.50) in the T2D women and Kyna and 3HKyn (r = −0.47) in the NG group. 3HKyn also correlated with QA in both the NG and T2D groups (r = −0.50; r = −0.38, respectively).

## 3. Discussion

### 3.1. The Relationship of Trp and Individual KP Metabolites with T2D

Our results showed that the obese T2D women had significantly higher Trp, Kyna, Kyna/QA ratio, and Kyna/3HKyn ratio values than the obese NG women. Our logistic regression analysis showed that the concentration of Trp and Kyna and values of Kyna/3HKyn ratio were most strongly associated with T2D occurrence, even after controlling for confounding factors. This combination of results may suggest that the Trp metabolic pathway shifts towards Kyna with an accompanying increase in Kyna/3HKyn ratio value in obese middle-aged women with T2D. The model with Trp level and Kyna/3HKyn ratio accounted for 20% of the variation in the presence of T2D. Additionally, we showed a different pattern of correlations between KP metabolites and metabolic factors in the NG and T2D obese women.

In previous case–control [13,17,18,19] and prospective studies [10,13,16,25], the most well-documented relationship was the one between T2D and Trp, while KP metabolites have been analyzed much less often. The results of these prospective and case–control studies are inconsistent and were performed across different populations. Moreover, the case–control studies were often performed on small groups without adjustment for BMI, age, or gender. The association of KP metabolites with T2D with larger cohorts of prediabetes and T2D patients was also analyzed. These studies were statistically controlled for confounding factors and were focused on an evaluation of the relationship between KP metabolites and the risk of diabetes-related disorders, e.g., acute myocardial infarction, cognitive impairment, and all-cause mortality [23,26]. In this study, we matched the groups to BMI and obesity categories as well as to other possibly confounding factors. It seems that the exclusion of these confounding factors, especially the difference in BMI between groups, is absolutely essential for a better evaluation of the relationship between T2D and KP in a study with a small sample size. 

Our results are partially in line with the results of other authors. Like them, we found higher concentrations of Trp [16] and Kyna [17,18,19,23] in T2D individuals. Some studies found no difference in Trp [18,19,23] or even lower Trp concentrations in diabetic patients, which was usually explained by the fact that Trp is metabolized to a greater extent in T2D patients than in nondiabetic subjects [17]. So far, only one study has been published that refers to obese European women from the ABOS cohort [13]. In this study, the authors did not show the differences in Trp, Kyn, KTR, Kyna, XA, or QA levels between obese women with and without diabetes [13]. 

Along with higher Kyna values, we observed a very weak tendency for 3HKyn concentrations to decrease, which was reflected in a significant increase in the Kyna/3HKyn ratio in the T2D obese women. Our finding that the Kyna/3HKyn ratio increases in T2D subjects has not been reported in the literature so far. As mentioned above, few studies have analyzed only 3HKyn, and three studies showed higher concentrations of it in T2D [17,19,23], whereas one did not show the difference in 3HKyn between diabetic and non-diabetic subjects [18]. To explain our results, we can only hypothesize that this increase in the Kyna/3HKyn ratio may be a consequence of the shift to Kyna or the enhancement of 3HKyn metabolism towards XA, which is recognized as a pro-diabetogenic metabolite [17,18].

Unexpectedly, we did not find any differences in Kyn and KTR values between the groups, which was also the case in the obese women from the ABOS cohort [13]. However, in some studies, higher levels of Kyn were observed in the T2D individuals [18,19]. The lack of differences in Kyn concentration may also be related to the fact that out of our T2D women, 79% were on metformin therapy. It was found that metformin might elevate the plasma levels of Trp and inhibit TDO expression in the liver, which may decrease the levels of Kyn in T2D women [27,28]. The higher activity of IDO-1 (KTR) is well-documented in obese subjects [12,13,14] and patients with cardiac events [16], and it is positively associated with inflammation [29], but the association between KTR and diabetes is less clear. A Norwegian study showed that KTR in the urine, but not in the plasma, was a strong predictor of T2D occurrence during 7 years of follow-up [10]. Other studies such as ours did not find an association between KTR and the occurrence of T2D [13,18]. It seems that the lack of difference in KTR may have partially been caused by the fact that our groups were matched for BMI and concentration of neopterin and CRP. Moreover, although the serum/plasma Kyn/Trp ratio (KTR) is commonly used to measure IDO activity, the enzyme activity indirectly calculated by the ratio of the product to the substrate is not perfect. Importantly, this ratio reflects not only IDO activity, but also TDO. Moreover, it was observed that Kyn undergoes almost complete reabsorption at very low plasma concentrations, but it is excreted to a greater extent via the renal route at the elevated serum levels that may occur in T2D. For this reason, a Norwegian prospective study concluded that urine KTR may potentially represent a more sensitive indicator of systemic Trp degradation than serum/plasma KTR [10].

### 3.2. The Relationship between Tryptophan and KP Metabolites with the Cardiometabolic Risk Factors

Along with Kyn and Kyna, Trp regulates glucose-induced insulin secretion (GSIS) in the pancreatic beta-cells and hepatic glucose production, and these processes may be altered when inflammation occurs [30]. The exposure of pancreatic islets to IFN-γ increased the levels of IDO and Kyn and potentiated GSIS by the normal islets [31]. It was also observed that moderate intakes of Trp affect fatty acid and triglyceride metabolism under physiological conditions and may be involved in the control of lipid metabolism in a variety of metabolic states [32]. Some human studies have shown that Trp and its metabolites are positively related to insulin resistance and other metabolic risk factors such as TG concentration and blood pressure [13,16,25]. In this study, we observed only a weak positive correlation between Trp and TG concentration, and this relationship was stronger in the T2D women.

More than 95% of dietary Trp is degraded in the KP, partially to QA, which is converted further to individual kynurenines, which are bioactive in different tissues. Kyn is the first KP metabolite. Kyn is an endogenous ligand for the human aryl hydrocarbon receptor (AHR), which regulates many genes and metabolic pathways, including immune response and glucose metabolism [31,33]. Recent animal studies have found that the Kyn-activated AHR caused an increase in body mass, adiposity, hepatic lipid deposition, and the disruption of glucose homeostasis, and can regulate GSIS in beta cells [34]. Human studies have indicated that Kyn correlated with BMI and decreased after bariatric surgery in T2D patients [35,36]. In this study, we did not observe significant correlations between Kyn and BMI or carbohydrate parameters, but we showed a weak correlation between Kyn and the activity of the liver enzyme ALT.

Kyn is further metabolized into the three downstream metabolites: Kyna, 3HKyn, and anthranilic acid (AA). We analyzed the first two because alterations were observed in them in T2D [3]. 3HKyn is recognized as being a pro-diabetogenic, pro-oxidant metabolite with a neurotoxicity function [4,19,37,38,39]. In this study, we only observed a weak positive tendency towards a correlation between 3HKyn and HOMA-IR and neopterin in the T2D women and positive correlations between BMI and CRP in the NG group.

Analysis of the correlations between kynurenines showed that 3HKyn correlated negatively with Kyna and QA, which may support the hypothesis that Kyna weakens the pathway toward 3HKyn synthesis, and that the formation of NAD from QA is impaired when the 3HKyn concentration increases in obese women. Moreover, the positive correlation between Trp and 3HKyn may suggest that KP is more shifted towards 3HKyn synthesis in obese T2D women with higher concentrations of Trp.

The role of increased levels of Kyna in T2D is still unclear. Kyna is recognized as an antagonist of the N-methyl-d-aspartate receptor (NMDAR) and as an endogenous agonist of the G-protein-coupled receptor 35 (GPR35) and AHR [2,40,41]. The effects of Kyna on NMDAR are associated with a neuroprotective function and may be associated with anti-diabetic properties through an increase in β-cell function and survival [42,43]. Kyna regulates GSIS and glucagon secretion [33,44]. Moreover, it was found that Kyna increases energy utilization by activating GPR35, which stimulates lipid metabolism and thermogenic and anti-inflammatory gene expression in adipose tissue. This suppresses weight gain in animals fed a high-fat diet and improves glucose tolerance [45]. All these animal studies support the theory that Kyna may have anti-diabetic properties. In humans, a positive relationship between Kyna and insulin resistance has been suggested [17,18,19], and the prooxidant properties of Kyna were observed in patients with proliferative diabetic retinopathy [19]. In this study, we only observed a very weak tendency towards a positive correlation between Kyna and HbA1c in the whole group of women and negative correlations between Kyna and BMI and CRP in the NG women.

The strength of this study is that our NG and T2D women were middle-aged and from a European population, as well as being matched for BMI, WC, concentration of inflammatory markers, physical activity, alcohol consumption, smoking habits, and menopausal status, and were also free of CKD, CVD, and acute inflammation (CRP < 10 mg/L). Because our groups differ in terms of age, eGFR, and GTP, we additionally adjusted our results to these parameters. Adjustment to confounding factors seems to be important as the concentration of Trp and its metabolites could be affected by many factors [6,12,13,14,21]. For example, the serum concentrations of KP metabolites were higher in men [21,23]; were lower in juvenile males; increased with age in adults, especially those above 60 years old [12,24,46,47]; and differed between ethnic groups, e.g., lower Kyna was observed in Caucasian women [6]. Additionally, liver and kidney function are associated with Trp metabolism [7,8].

As a limitation of the present study, we have to mention the small sample size. For this reason, we could not perform multifactor adjustments of our analyses. Another limitation is the lack of determination of the remaining metabolites of the KP, especially XA. Additionally, we did not measure the plasma concentrations of vitamin B-6 (pyridoxal 5′-phosphate; P5P), but we only hypothesize that the concentration of P5P was not statistically different between the groups since both of them presented similar inflammation status and dietary patterns. Moreover, our study does not allow us to establish the biological mechanisms or causality responsible for the relationship between circulating KP metabolites and glycemic status. We only showed the statistical relationships between these parameters and suggested some potential mechanisms based on the literature review. Because we analyzed a selected group of obese women, our results are not fully representative of the general population. Further investigation is warranted and will include a larger cohort and additional KP metabolites.

## 4. Materials and Methods

### 4.1. Participants

The study included 128 obese Caucasian women between the ages of 41 and 60. These women were selected from a group included in a previous cardiometabolic risk factor study performed at the Department of Laboratory Medicine of the Nicolaus Copernicus University in Bydgoszcz, Poland, in the years from 2016 to 2019. The exclusion criteria were as follows: history of diabetes mellitus type 1, history of cardiovascular disease (CVD), and severe or moderate chronic kidney disease (CKD). All the women were obese with a BMI ≥ 30 kg/m^2^ and had serum C-reactive protein (CRP) < 10 mg/L, thyroid stimulating hormone (TSH) < 4.94 μIU/mL, and eGFR > 60 mL/min/1.73 m^2^. Age, smoking status, alcohol consumption habits and physical activity, menopausal status, medical history, and medications were investigated using a medical questionnaire. Height (cm), weight (kg), waist circumferences (WC, cm), and systolic (SBP) and diastolic (DBP) blood pressures were measured using standard methods. Postmenopausal status was defined as the permanent cessation of menstruation for at least 12 months [48]. Smoking status was classified as current smoker, ex-smoker, and non-smoker. Physical activity and alcohol consumption were classified as never (0 point), sporadically (1 point), more than once a month (2 points), once a week (3 points), 3 to 4 times a week (4 points), and every day (5 points). Obesity categories were classified as class 1: BMI of 30 to <35; class 2: BMI of 35 to <40; class 3: BMI ≥ 40 kg/m^2^. T2D was diagnosed according to a fasting glucose level in plasma ≥ 126 mg/dL (≥7.0 mmol/L) repeated on two consecutive days, or HbA1c above or equal to 6.5% (48 mmol/mol), or self-reported, physician-diagnosed diabetes and the use of glucose-lowering medications for diabetes. The normoglycemic state was indicated by a fasting glucose level in plasma < 5.6 mmol/L (100 mg/dL) [49]. The study participants continued their normal omnivorous diet regimen. None of them followed an elimination or high-protein diet. Dietary supplements, including vitamin B complex, Trp, 5-hydroxytryptophan (5-HTP), and protein supplements (e.g., whey, casein) were not used either.

The obese women were divided into two groups: (1) a normoglycemic group (NG; *n* = 65): women with normal fasting glucose, without a history of diabetes, and (2) a T2D group (T2D; *n* = 63).

### 4.2. Blood Sampling and Laboratory Analyses

Fasting venous blood samples were collected in the early morning for all parameters. The parameters of triglycerides (TG), HDL-cholesterol (HDL-C), creatinine, CRP, alanine aminotransferase (ALT), gamma glutamyltransferase (GGT), insulin, and TSH were measured in the serum, while the plasma was used for glucose measurement, and EDTA whole blood was used to determine glycated hemoglobin (HbA1c) as previously described [50]. The HOMA-IR value was calculated [51]. The estimated glomerular filtration rate (eGFR) was calculated using the CKD-EPI creatinine equation [52]. Hypertriglyceridemia was defined as triglycerides ≥ 150 mg/dL (1.7 mmol/L) or the use of lipid-lowering medications due to a previous diagnosis [53]. Trp and KP metabolites were analyzed in previously deep-frozen serum samples using ultra-high-performance liquid chromatography coupled with electrospray ionization/triple quadrupole mass spectrometry (UHPLC-ESI-MS/MS) as described by Sadok et al. [5] with some modifications (Supplementary Materials).

The concentrations of Trp and individual KP metabolites were used to calculate the following ratios: the Kyn/Trp ratio (KTR), which reflects IDO activity; the Kyna/Kyn ratio (KAT), which reflects KAT activity; the 3HKyn/Kyn ratio (KMO), which reflects KMO activity; and the Kyna/3HKyn ratio and the Kyna/QA ratio, which reflect the imbalance between kynurenines.

The study protocol was approved by the Bioethics Committee at the Nicolaus Copernicus University in Torun and Collegium Medicum in Bydgoszcz, and written informed consent was obtained from all the study participants (approval number KB 392/2018).

### 4.3. Statistical Analysis

The data were presented as means ± standard deviation SD (Gaussian distribution) or medians and 25th and 75th percentiles (non-Gaussian distribution). The Shapiro–Wilk test was applied to test the Gaussianity. The variables were compared using Student’s t-test (Gaussian) and the Mann–Whitney U test (non-Gaussian). To test for the significance of difference between the percentages, we used the chi-squared test. Parameters with non-Gaussian distribution were normalized by natural log transformation. The Spearman rank correlation coefficients were computed. Logistic regression based on variables transformed to z-scores was applied. Goodness of fit was evaluated by Hosmer and Lemeshow chi-square. Trp and individual KP metabolites and their ratios were included separately in logistic regression models as independent variables, with T2D as the dependent variable. All logistic regression models were adjusted for age, BMI, eGFR, GGT, and hypertriglyceridemia. The significance of the logistic models was tested by the Wald chi-squared statistic. To correct *p*-values, the Benjamini–Hochberg procedure was applied to risk factor results to reduce the potential for type 1 error. During sample size determination, a significance level of 0.05 was applied for parametric two-tailed Student’s t-tests with a power level of 0.9. For nonparametric Mann–Whitney tests, the minimum sample size required was increased by 15%. According to the pilot study, we calculated that enrolment of 65 women in the NG group and 63 women in the T2D group would provide a power of 94% to show a significant difference in Trp concentrations. We decided to obtain such a high power to be able to perform credible multivariable analyses. The level of statistical significance was set as 0.05 (Statistica 13.3, StatSoft or MedCalc statistical software).

## 5. Conclusions

T2D occurrence is mainly associated with an increase in Trp and Kyna concentrations with an accompanying increase in the Kyna/3HKyn ratio value. The different patterns of correlations between kynurenines and metabolic factors in the obese NG and T2D women are mostly reflected in the stronger relationship between BMI and KP metabolites in the NG women. These results may support the hypothesis that a modification in the kynurenine pathway may be a potential therapeutic target for diabetes in obese women. Moreover, KP metabolites may serve as a potential diagnostic tool for cardiometabolic disturbances and T2D in obese women. However, future studies are required to verify our findings in a larger group of obese middle-aged women.

## Figures and Tables

**Table 1:** The basal characteristics of obese normoglycemic (NG) and T2D women.

Parameters	NG Women *n* = 65	T2D Women *n* = 63	*p*
Age (years)	50.0 ± 5.0	53.2 ± 5.0	<0.001
BMI (kg/m^2^)	36.5 ± 6.2	36.6 ± 6.3	0.91
WC (cm)	106.8 ± 16.4	107.7 ± 13.9	0.76
Glucose (mmol/L)	5.2 (4.8–5.4)	7.0 (5.9–8.1)	<0.0001
HDL-C (mmol/L)	1.4 (1.2–1.7)	1.2 (1.0–1.4)	0.001
TG (mmol/L)	1.3 (0.9–1.6)	1.7 (1.3–2.2)	0.0001
ALT (U/L)	18.7 (14.2–23.8)	22.7 (17.8–30.4)	0.009
GGT (U/L)	20.7 (14.6–25.8)	25.2 (18.9–33.7)	0.011
Creatinine (mg/dl)	0.76 (0.72–0.88)	0.81 (0.73–0.89)	0.23
eGFR (mL/min/1.73 m^2^)	91.9 (80.2–96.4)	83.1 (71.7–92.4)	0.04
CRP (mg/L)	2.3 (1.0–3.9)	3.3 (1.3–5.9)	0.08
HbA1c (mmol/mol)	37.0 (34.0–38.0)	45.0 (42.0–58.0)	<0.0001
Insulin (µIU/mL)	9.0 (6.7–12.1)	10.5 (5.8–15.0)	0.29
TSH (mIU/L)	1.48 (1.14–2.10)	1.34 (1.01–2.05)	0.31
HOMA-IR	2.0 (1.6–2.9)	3.3 (1.9–5.5)	0.0005
Neopterin (nmol/L)	7.0 (5.0–8.9)	7.3 (4.2–10.4)	0.61
SBP (mmHg)	130 (120–140)	130 (120–140)	0.69
DBP (mmHg)	80 (80–90)	80 (80–85)	0.45
Lipid-lowering treatment (%)	16	43	0.0008
Hypertension treatment (%)	33	62	0.01
Metformin treatment (%)	0	76	<0.0001
Menopausal status (% postmenopausal)	50	62	0.11
Physical activity (% never or sporadically)	32	35	0.72
Alcohol consumption (% never or sporadically)	94	95	0.80
Smoker (% current)	12	19	0.27
Obesity categories 1/2/3 (%)	54/18/28	45/31/24	0.67

Means ± SD or medians (25th and 75th percentiles) or %.

**Table 2:** The values of tryptophan and KP metabolites in obese NG and T2D women.

Parameters	NG Women *n* = 65	T2D Women *n* = 63	*p*	*p* *
Trp (µmol/L)	55.0 (50.0–58.5)	59.2 (51.6–70.8)	0.009	0.033
Kyn (µmol/L)	1.39 (1.20–1.84)	1.53 (1.17–1.86)	0.76	0.76
Kyna (µmolL)	0.097 (0.078–0.117)	0.110 (0.093–0.128)	0.01	0.033
3HKyn (µmol/L)	0.30 (0.21–0.44)	0.28 (0.18–0.41)	0.04	0.08
QA (µmol/L)	0.16 (0.10–0.37)	0.15 (0.10–0.24)	0.36	0.40
KTR	24.9 (20.6–33.6)	22.6 (18.8–35.2)	0.30	0.38
KMO	0.20 (0.13–0.32)	0.20 (0.10–0.31)	0.22	0.31
KAT	0.060 (0.048–0.088)	0.072 (0.052–0.097)	0.11	0.18
Kyna/QA	0.54 (0.29–0.87)	0.72 (0.45–1.13)	0.02	0.05
Kyna/3HKyn	0.32 (0.14–0.48)	0.47 (0.34–0.77)	0.008	0.033

Means ± SD or medians (25th and 75th percentiles); Trp (tryptophan); Kyn (kynurenine); Kyna (kynurenic acid); 3HKyn (3-hydroxykynurenine); QA (quinolinic acid); KMO (3HKyn/Kyn ratio); KAT (Kyna/Kyn ratio); *p* *—corrected; *p*-value after applying Benjamini–Hochberg correction.

**Table 3:** The Spearman rank correlation coefficients in all the women.

Parameter	Trp	Kyn	Kyna	3HKyn	QA	KTR	KMO	KAT	Kyna/ QA	Kyna/ 3HKyn
Age	-	-	-	-	-	-	-	-	-	-
BMI	0.21 *	-	−0.18 *	0.31 ^$@^	−0.34 ^$@^	-	0.23 *	−0.20 *	0.23 *^@^	−0.26 ^#@^
WC	-	-	−0.22 *	0.22 *	−0.29 ^#@^	-	0.10 *	−0.18 *	0.18 *	−0.22 *
Glucose	-	-	-	-	-	-	-	-	-	-
HOMA-IR	0.18 *	-	-	-	-	-	-	-	-	-
HDL-C	-	-	-	-	-	-	-	-	-	-
TG	0.30 ^$@^	-	-	-	−0.28 ^#@^	−0.21 *	-	-	0.28 ^#@^	-
ALT	-	0.27 ^#@^	-	-	-	-	-	−0.25 ^#@^	-	-
GGT	0.18 *	0.24 ^#^	−0.19 *	-	-	-	-	−0.31 ^$@^	-	-
HbA1c	0.18 *	-	0.23 *	-	-	-	-	0.20 *	0.19 *	0.22 *
CRP	-	-	-	-	−0.19 *	-	-	-	-	-
Neopterin	-	-	-	-	-	-	-	-	-	-
Creatinine	0.22 *	-	0.20 *	-	−0.26 ^#@^	-	-	-	0.33 ^$@^	-
eGFR	−0.24 ^#^	-	−0.18 *	-	0.28 ^#@^	-	-	-	−0.35 ^$@^	0.20 *

(-) not statistically significant before correction; * *p* < 0,05; ^#^ *p* < 0.01; ^$^ *p* < 0.001; ^@^—statically significant after applying Benjamini–Hochberg correction.

**Table 4:** The associations between Trp and KP metabolites with T2D in logistic regression models.

Independent Variables	Odds Ratios (95% CI) per 1SD Increase in Value of Independent Variable					
	Model Unadjusted	Model Adjusted for BMI	Model Adjusted for Age	Model Adjusted for eGFR	Model Adjusted for GGT	Model Adjusted for Hipertg&
**Trp**	1.81 * (1.13–2.88)	1.81 * (1.12–2.90)	1.68 * (1.02–2.74)	1.78 * (1.09–2.91)	1.73 * (1.11–3.0)	1.75 * (1.06–2.88)
**Kyna**	2.50 ^#^ (1.28–4.70)	2.37 ^#^ (1.24–4.53)	2.48 ^#^ (1.27–4.84)	1.99 * (1.07–3.71)	2.51 ^#^ (1.29–4.92)	2.27 * (1.89–4.33)
**Kyna/** **3HKyn**	1.92 ^#^ (1.21–2.70)	1.83 ^#^ (1.19–2.80)	1.90 ^#^ (1.23–2.92)	1.75 ^#^ (1.15–2.65)	1.91 ^#^ (1.25–2.98)	1.66 * (1.10–2.51)
**Kyna/QA**	1.81 * (1.08–3.04)	1.82 * (1.08–3.08)	1.69 (0.98–2.91)	1.62 (0.94–2.79)	1.81 * (1.07–2.99)	1.69 (0.99–2.87)

& hypertriglyceridemia: TG ≥ 150 mg/dL and/or lipid-lowering treatment; CI (confidence interval). All models achieved statistical goodness of fit. * *p* < 0.05; # *p* < 0.01. Nagelkerke R-squared of unadjusted models: Trp 0.073; Kyna 0.075; 3HKyn 0.052, Kyna/3HKyn ratio 0.083, Kyna/QA ratio 0.061; the statistically significant associations remained significant after applying Benjamini–Hochberg correction.

**Table 5:** The Spearman rank correlation coefficients between Trp, KP metabolites, and metabolic factors according to glycemic status.

Parameter	NG Correlation Coefficients/*T2D Correlation Coefficients*									
	Trp	Kyn	Kyna	3HKyn	QA	KTR	KMO	KAT	Kyna/ QA	Kyna/ 3HKyn
**BMI**	0.29/-	-/-	−0.43 ^@^/-	0.66 ^@^/-	−0.60 ^@^/-	−0.34 ^@^/-	0.60 ^@^/-	−0.29/-	0.41 ^@^/-	−0.63 ^@^/-
**WC**	0.24/-	-/-	−0.40 ^@^/-	0.46 ^@^/-	−0.50 ^@^/-	−0.36 ^@^/-	0.48 ^@^/-	−0.34 ^@^/-	0.32 ^@^/-	−0.48 ^@^/-
**Glucose**	-/-	-/-	−0.26/-	-/-	-/-	-/-	-/-	-/-	-/-	
**HOMA-IR**	-/-	-/-	-/-	-/***0.30***	-/-	-/-	-/-	-/-	-/-	-/−***0.28***
**HDL-C**	-/-	-/-	-/-	-/-	-/-	-/-	-/-	-/-	-/-	
**TG**	-/***0.29***	-/-	−0.24/-	0.31 ^@^/-	−033 ^@^/-	-/−***0.27***	0.26/-		-/-	−0.29/-
**ALT**	-/-	0.27/ ***0.28***	-/-	-/-	-/-	-/-	-/-	−0.33/ *−**0.26***	-/-	-/-
**GGT**	-/-	0.25/ ***0.30***	-/*−**0.32***	-/-	-/-	-/-	-/-	−0.35 ^@^/ *−**0.40***	-/-	-/-
**HbA1c**	-/-	-/-	-/-	-/-	-/-	-/-	-/-	-/-	-/-	-/-
**CRP**	-/-	-/-	−0.34 ^@^/-	0.35 ^@^/-	−0.30/-	-/-	0.34 ^@^/-	-/-	-/-	−0.37 ^@^/-
**Neopterin**	-/-	-/-	-/-	-/***0.32***	-/-	-/-	-/-	-/-	-/***0.32***	-/*−**0.26***
**Creatinine**	0.29/-	-/-	-/-	-/-	−0.27/-	-/-	-/-	-/-	0.33 ^@^/***0.29***	-/-
**eGFR**	−0.26/-	-/-	-/-	-/-	0.29/-	-/-	-/-	-/-	−0.32 ^@^/ −***0.28***	-/-

(-) not statistically significant before correction; NG (normoglycemic women); T2D (type 2 diabetes women). ^@^—Statically significant after applying Benjamini–Hochberg correction; text in bold/italic means T2D correlation coefficients.

## Data Availability

The data presented in this study are available on request from the corresponding author. The data are not publicly available due to privacy.

## Supplementary Materials

The following supporting information can be downloaded at https://www.mdpi.com/article/10.3390/metabo12060492/s1, File: Quantitative analysis of Trp and KP metabolites in serum samples and preparation of serum and calibration standards for UHPLC-ESI-MS/MS analysis; Table S1: DMRM acquisition parameters DMRM acquisition parameters.
