# Supplementary material for: The Kynurenine Pathway in Obese Middle-Aged Women with Normoglycemia and Type 2 Diabetes
Source: Metabolites. 2022 May 29;12(6):492. doi: 10.3390/metabo12060492 (PMC9230031; doi:10.3390/metabo12060492)
Supplement: Supplementary file 1 [file metabolites-12-00492-s001.zip › metabolites-1723478-SM.pdf]

Manuscript title: The kynurenine pathway in obese middle-aged women with normoglycemia and type 2 diabetes

Justyna Kubacka, Magdalena Staniszewska, Ilona Sadok, Grazyna Sypniewska, Anna Stefanska

Supplementary Materials

Reagents

In the chromatographic analysis we used methanol (hypergrade), ammonium acetate (LC-MS grade), HCl, and dimethyl sulfoxide (DMSO) from Merck (Darmstadt, Germany). Trp, KP metabolites, bovine serum albumin (BSA), trichloroacetic acid (TCA), and activated charcoal were purchased from Sigma-Aldrich (St Louis, MO, USA). Deuterated $^{2}H_{3}$-3HKyn was obtained from Buchem B.V. (Apeldoorn, Netherlands). Deuterated $^{2}H_{3}$-QA, $^{2}H_{5}$-Trp, and $^{2}H_{5}$-Kyna were from Trc Canada (Toronto, Canada). The stock solution (1 g/L) of Trp, $^{2}H_{5}$-Trp, Kyn, Kyna, and $^{2}H_{5}$-Kyna was prepared in DMSO, QA and $^{2}H_{3}$-QA in methanol, and 3HKyn and $^{2}H_{3}$-3HKyn in water acidified with HCl (pH 2.5). Working solutions were prepared daily at intermediate concentrations by dilution in methanol.

Quantitative analysis of Trp and KP metabolites in serum samples

UHPLC-ESI-MS/MS measurements were performed using instrumentation, software, and analytical columns as described in [1] for the serum samples. Herein, the analytes and corresponding stable isotope labeled standards (SILS) were separated using a mobile phase consisting of solvent A (5 mmol/L ammonium acetate in water) and solvent B (methanol). The gradient program was as follows: 5% to 40% B (0-4.5 min); 40% to 90% B (4.5-6.5 min); 90% B (6.5-8.0 min); 90% to 5% B (8.0-9.0 min); 5% B (9.0-12.0 min); post run: 2 min. The mobile phase rate and the injection volume were 0.25 mL/min and 2 μL, respectively. All samples were analyzed in triplicate. The mass detector settings were as follows: nebulizer – 35 psi; gas temperature – 300 °C; gas flow – 5.5 L/min; sheath gas temperature – 300 °C; sheath gas flow –- 7 L/min; spray voltage – 4000 V. The ions were monitored using a Dynamic Multiple Reaction Monitoring (DMRM) mode. The MRM transitions and other acquisition parameters are collected in Table 1. For quantitative analysis, matrix-matched calibration curves prepared with SILS addition were used.

Table S1. DMRM acquisition parameters

			Transitions [m/z]				
**Compound**	**SILS**	**Retention time [min]**	**Precursor ion**	**Product ion**	**Fragmentor [V]**	**Collision energy [eV]**	**Delta retention time [min]**
QA	$^{2}H_{3}$-QA	1.25	168	150	60	8	3
		1.25	168	124	60	8	3
$^{2}H_{3}$-QA	-	1.25	171	153	60	8	3
		1.25	171	127	60	8	3
3HKyn	$^{2}H_{3}$-3HKyn	1.52	225	208	80	8	3
		1.52	225	162	80	10	3
$^{2}H_{3}$-3HKyn	-	1.52	228	211	80	6	3
		1.52	228	111	80	12	3
Kyn	$^{2}H_{5}$-Trp	2.17	209	192	100	8	3
		2.17	209	174	100	10	3
Kyna	$^{2}H_{5}$-Kyna	3.9	190	172	100	10	3
		3.9	190	144	100	20	3
$^{2}H_{5}$-Kyna	-	3.9	195	177	120	12	3
		3.9	195	149	120	20	3
Trp	$^{2}H_{5}$-Trp	4.05	205	188	110	8	3
		4.05	205	159	110	10	3

$^{2}H_{5}$-Trp	-	4.05	210	192	100	4	3
		4.05	210	150	100	16	3

Preparation of serum and calibration standards for UHPLC-ESI-MS/MS analysis

The serum samples (90 µL) were transferred to conical tubes, fortified with a 10 µL SILS mixture (containing: 69.0 µmol/L $^{2}H_{3}$-3HKyn; 48.0 µmol/L $^{2}H_{3}$-QA; 13.25 µmol/L $^{2}H_{5}$-Kyna; 29.4 µmol/L $^{2}H_{5}$-Trp) and vortexed well. To remove the proteins from the sample, 50 µL of 15% (w/v) TCA was added. The solutions were vortexed well and double centrifuged at 16,000 ×g for 15 min at 4 °C (5415R Centrifuge, Eppendorf, Germany). The clear supernatants were transferred to chromatographic insert vials and immediately analyzed using the UHPLC-ESI-MS/MS method. For Trp quantification, the serum samples were fortified with $^{2}H_{5}$-Trp (0.29 mmol/L) and processed as described above. The supernatants obtained before analysis were 100 times diluted with an aqueous solution of 5 mmol/L ammonium acetate.

A matrix-matched calibration standard series was built using a charcoal-purified BSA solution (prepared as described in[1]) spiked with analytes at different concentration levels with constant amounts of SILS, and further processed as described above.

References

1. Sadok, I.; Jędruchniewicz, K.; Rawicz-Pruszyński, K.; Staniszewska, M. UHPLC-ESI-MS/MS Quantification of Relevant Substrates and Metabolites of the Kynurenine Pathway Present in Serum and Peritoneal Fluid from Gastric Cancer Patients—Method Development and Validation. Int. J. Mol. Sci. 2021, 22, 6972. https://doi.org/10.3390/ijms22136972
